# Enoxaparin dosing for venous thromboembolism prophylaxis in hospitalized underweight adult patients: a retrospective cohort study

**DOI:** 10.1186/s12959-025-00716-w

**Published:** 2025-05-07

**Authors:** Salam Ibrahim Abou Safrah, Mohamed Omar Saad, May Alasmar, Fatima Ashfaq Butt, Somaya Khaled Koraysh

**Affiliations:** 1 Pharmacy Department, Hamad General Hospital, Doha, Qatar; 2 Pharmacy Department, Al-Wakra Hospital, Doha, Qatar; 3 Pharmacy Department, Hamad Bin Khalifa Medical City, Doha, Qatar

**Keywords:** Enoxaparin, Dose, Prophylaxis, Venous thromboembolism, Deep vein thrombosis, Body mass index, Weight

## Abstract

**Background:**

Enoxaparin is commonly used for venous thromboembolism (VTE) prophylaxis in adult hospitalized patients. Although anti-Xa levels are inversely related to body weight, limited studies evaluated clinical outcomes of dose reduction in the underweight population.

**Objective:**

To compare the incidence of bleeding and VTE in underweight patients receiving reduced doses of enoxaparin (< 40 mg daily) versus the standard dose (40 mg daily) for VTE prophylaxis.

**Methods:**

This was a multicentre retrospective cohort study at Hamad Medical Corporation in Qatar. We included hospitalized patients with a total body weight ≤ 57 kg or body mass index (BMI) ≤ 18.5 kg/m^2^ who received prophylactic enoxaparin for at least 48 h. The outcomes were bleeding, VTE, and composite unfavourable outcome (bleeding or VTE). Inverse-probability-of-treatment weighting (IPTW) was used to adjust for confounding.

**Results:**

We identified 1,130 eligible patients, of whom 124 patients (11%) received the reduced dose, and 1,006 patients (89%) received the standard dose. Bleeding occurred in one patient (0.8%) of the reduced dose group compared to 15 patients (1.5%) in the standard dose group (*p* > 0.99), VTE occurred in two patients (1.6%) in the reduced dose group compared to four patients (0.4%) in the standard dose group (*p* = 0.13). In the IPTW analysis, there was no significant difference in overall bleeding (odds ratio (OR) 1.4, 95% CI 0.18–10.75, *p* = 0.74), VTE (OR 0.3, 95% CI 0.05–1.81, *p* = 0.19), or the composite unfavourable outcome (OR 0.74, 95% CI 0.2–2.75, *p* = 0.66).

**Conclusion:**

There is no significant difference in the incidence of bleeding or VTE between the reduced dose and the standard dose of enoxaparin for VTE prophylaxis in underweight adult patients. Due to the low event rates in both groups, larger studies are required to delineate any differences between the two dosing strategies.

**Supplementary Information:**

The online version contains supplementary material available at 10.1186/s12959-025-00716-w.

## Introduction

Venous thromboembolism (VTE) is a preventable cause of morbidity and mortality in hospitalised patients [1]. It is defined by either deep venous thrombosis (DVT) or pulmonary embolism (PE) [1]. The incidence of VTE can reach up to 11% in high-risk hospitalised medical patients and as high as 30% in surgical patients; therefore, measures to prevent VTE are essential components of in-hospital patient care [2]. Risk factors for VTE include active cancer, reduced mobility, recent trauma or surgery, advanced age, heart failure, respiratory failure, acute myocardial infarction (MI), ischemic stroke, acute infection, and obesity [1, 3].

Pharmacologic prophylaxis with anticoagulants is recommended for high-risk hospitalised patients to reduce the incidence of VTE and its consequences [1]. Low-molecular-weight heparins (LMWH), such as enoxaparin, are preferred over unfractionated heparin (UFH) in patients with normal kidney function for their convenience in dosing and safety profile [1]. The standard prophylactic dose of enoxaparin is 40 mg subcutaneously (SC) once daily for normal-weight patients [4]. However, the optimum dose for underweight patients is not well established.

Previous studies suggest that low body weight is associated with increased bleeding risk in patients receiving standard prophylactic doses of enoxaparin for VTE prophylaxis [5, 6]. This can be attributed to higher anti-factor Xa levels in underweight patients than in normal weight control subjects [7] due to differences in the volume of distribution and renal clearance [8]. However, the manufacturer recommends individualised clinical monitoring of patients with low body weight but does not specifically recommend a dose reduction due to the lack of evidence in this population [4]. In addition, practice guidelines do not make specific recommendations for dose adjustments in underweight patients [1, 4]. The 2018 American Society of Haematology (ASH) guidelines on VTE prophylaxis recommend further research regarding dose adjustment in underweight patients [9].

Recent findings further emphasize the need for personalized anticoagulation strategies. A cohort study observed that underweight elderly inpatients on thromboprophylaxis frequently achieve supratherapeutic anti-Xa levels, underscoring the importance of individualized dosing strategies [10]. Smaller studies have suggested that lower doses of enoxaparin may be sufficient to achieve adequate anti-Xa levels for VTE prophylaxis in underweight patients [11, 12]. However, larger clinical studies have not consistently shown a significant reduction in bleeding risk with dose reduction [13–17], likely due to limitations such as small sample sizes and inadequate adjustment for confounding factors. In this study, we aimed to compare the incidence of bleeding and VTE in hospitalized underweight adult patients receiving reduced doses of enoxaparin (< 40 mg daily) versus the standard dose (40 mg daily) for VTE prophylaxis.

## Materials and methods

### Study design and participants

This multicentre retrospective cohort study was conducted at Hamad Medical Corporation (HMC). We included hospitalised adult patients (age ≥ 18 years at admission) who received enoxaparin for the prevention of VTE for at least 48 h, stayed in the hospital for more than 48 h, and had a body mass index (BMI) of less than 18.5 kg/m^2^ or total body weight (TBW) ≤ 57 kg. We excluded patients admitted with suspected VTE or bleeding, who were already on oral anticoagulants, or who had a creatinine clearance (CrCl) < 30 mL/min. Patients who met these criteria between January 1, 2016, and December 31, 2019, were included.

Patients were categorised into the standard dose group if they received enoxaparin 40 mg per day or the reduced dose group if they received less than 40 mg per day. We considered only the first hospital admission where each patient met the inclusion criteria. We collected patient demographics, comorbidities, enoxaparin dosing regimens, concomitant medications, estimated creatinine clearance by Cockcroft-Gault equation using actual body weight, individual components of Padua VTE risk score (active cancer, previous VTE, reduced mobility, known thrombophilic condition, recent trauma and/or surgery within one month, elderly age (≥ 70 years), heart or respiratory failure, acute MI or ischemic stroke, acute infection or rheumatologic disorder, obesity (BMI ≥ 30 kg/m^2^), and ongoing hormonal treatment), and details of bleeding and VTE events. Relevant data were extracted from the electronic medical records and then verified by manual chart review.

### Outcomes

The outcomes were bleeding - either major or clinically relevant non-major bleeding (CRNMB), and VTE. Additionally, we evaluated a composite unfavourable outcome defined as the incidence of bleeding or VTE. Major bleeding was defined as fatal bleeding, haemoglobin drop by ≥ 2 g/dL, the requirement of transfusion ≥ 2 units of packed red blood cells or whole blood in 24 h, or bleeding occurring in any critical organ (retroperitoneal, intracranial, intraspinal, intraocular, intra-articular, pericardial, or intramuscular with compartment syndrome) [13]. CRNMB was defined as any bleeding that did not meet the criteria for major bleeding but required medical intervention by a healthcare professional or led to an increased level of care. Haemoglobin decreases ≥ 2 g/dL that occurred within the first 24 h of admission in patients who received 30 mL/kg of intravenous fluids were considered dilutional and not a bleeding event. VTE was defined as a diagnosis of DVT [including proximal or distal] or PE by a Doppler ultrasound scan or a CT-angiography scan, respectively. All patient encounters underwent a manual chart review to confirm VTE and bleeding. The outcome follow-up period was considered from the first dose until 24 h after the last dose of enoxaparin.

### Statistical analysis

Demographic and clinical characteristics were described using frequencies and percentages for categorical variables and medians and ranges for continuous variables. Demographic and clinical characteristics were grouped by enoxaparin dosing regimen (standard versus reduced) and compared using Fisher's exact test for categorical variables and Wilcoxon rank-sum for continuous variables.

The incidences of major bleeding, CRNMB, and VTE were calculated overall and by the dosing regimen and were compared using Fisher’s exact test. We planned to adjust the analysis of each outcome for predefined potential confounders; however, this was not possible due to the small number of bleeding and VTE events. We used inverse probability of treatment weighting (IPTW) to adjust the analysis of the composite outcome for baseline differences in Padua VTE risk score, critical illness, and history of bleeding. Calculated weights were stabilised by multiplying them by the marginal probability of receiving the given enoxaparin regimen. The balance of potential confounders was evaluated using standardised differences between the two groups. Logistic regression using weighted observations and a robust variance estimator was used to estimate the odds ratios (OR) of the outcomes with their 95% confidence intervals (CI). To account for interruptions in therapy and switching between regimens, the dosing group was modelled as a time-varying exposure in a survival analysis with death modelled as a competing risk. Patients were censored at the time of the first event occurrence (either bleeding or VTE). This approach ensured that patients who developed a VTE were not followed for bleeding outcomes thereafter, and vice versa. Sub-distribution hazard ratios (sHR) with their 95% CIs were used to compare the two dosing strategies. The statistical significance level was set at *P* < 0.05 for all tests. Analyses were conducted using Stata 17.

### Ethical approval

The study was approved by Hamad Medical Corporation (HMC) Institutional Review Board MRC- 01–22–477.

## Results

**Fig. 1:**
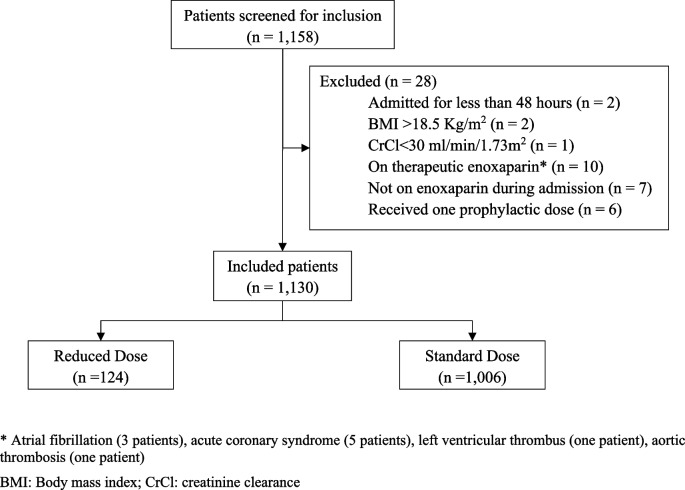
Flow diagram of patient selection, showing the total cohort screened, reasons for exclusion, and the final study population. BMI: Body mass index; CrCl: creatinine clearance. *atrial fibrillation (3 patients), acute coronary syndrome (5 patients), left ventricular thrombus (one patient), aortic thrombosis (one patient)

Out of 1,158 screened patients for eligibility, 1,130 patients met the inclusion/exclusion criteria. The most common reason for exclusion was the use of therapeutic enoxaparin for the treatment of atrial fibrillation, left ventricular thrombus, or acute coronary syndrome. Additional exclusions were due to factors such as brief hospital admission (less than 48 h), BMI > 18.5 kg/m^2^, impaired renal function (creatinine clearance < 30 ml/min/1.73 m^2^), not receiving enoxaparin during admission, or receiving only one prophylactic dose of enoxaparin (Fig. 1)*.*

Overall, 1,130 patients were included in the analysis: 124 patients (11%) received a reduced enoxaparin dose, and 1,006 patients (89%) received the standard prophylactic dose of enoxaparin (40 mg). Among patients who received a reduced dose, 65 patients received 30 mg, 58 patients received 20 mg, and one patient received 15 mg. Overall, most patients were middle-aged. More patients were females in the reduced dose group (75%) compared to the standard dose group (50.6%) (*P* < 0.0001). The main reason for admission was acute infection (49.2%), followed by major trauma or surgery (26.7%). The median Padua score was four. More patients in the reduced dose group had respiratory failure upon admission compared to the standard dose group (9.7% vs 2.3%, *P* < 0.001). Patients who were bedridden or paralyzed were more common in the reduced dose group as compared to the standard group (23.4% vs 10.5%, *P* < 0.001). Similarly, critical illness was more common in the reduced dose group (16.1% vs 6.9%, *P* < 0.001) (Table 1). A total of 229 patients received NSAIDs, and 165 patients received antiplatelets while receiving enoxaparin (Table 2). Other methods for VTE prophylaxis included intermittent pneumatic compression (IPC), which was used concurrently with enoxaparin in 18.5% and 16.1% of patients in the reduced and the standard-dose groups, respectively. The median (IQR) total duration of enoxaparin therapy was 4 (3–9) days in the reduced dose group and 4.8 (3–9.5) days in the standard dose group.

**Table 1:** Baseline characteristics of underweight patients who received enoxaparin for venous thromboembolism prophylaxis

Characteristic	Reduced dose (*n* = 124)	Standard dose (*n* = 1,006)	*P* value
Female sex^a^	93 (75)	509 (50.6)	< 0.001
Age [years]^b^	29.8 (24.5- 56)	36.5 (27.6–54.1)	0.041
Weight [Kg]^b^	45 (39–48.8)	51 (46–54)	< 0.001
Height [cm]^b^	153.7 (148–159.5)	158 (153–165)	< 0.001
BMI calculated (Kg/m^2^)^b^	18.1 (16.2- 20.8)	19.6 (17.8–21.8)	< 0.001
CrCl [mL/min]^b^	108 (65–131.9)	99.5 (73.7–125.7)	0.49
INR on enoxaparin initiation^b^	1.1 (1–1.2)	1.1 (1–1.2)	0.87
Platelets^b^	247 (194–319)	268 (206- 348)	0.13
D-dimer^b^	3.5 (0.8- 7.5)	2.1 (0.8–4.4)	0.34
Hgb^b^	11.2 (9.9–12.4)	11.7 (10.4–13.4)	0.001
Padua score^b^	4 (4–5)	4 (4–5)	0.76
History of DVT/PE^a^	0 (0)	12 (1.2)	0.38
Heart Failure^a^	3 (2.4)	37 (3.7)	0.61
Acute MI or stroke < 1 month^a^	4 (3.2)	65 (6.5)	0.23
Respiratory failure^a^	12 (9.7)	23 (2.3)	< 0.001
Acute infection at admission^a^	66 (53.2)	495 (49.2)	0.45
History of bleeding^a^	4 (3.2)	26 (2.6)	0.56
Critical illness^a^	20 (16.1)	69 (6.9)	< 0.001

*Abbreviations:* Reduced dose: enoxaparin < 40 mg SC daily, Standard dose: enoxaparin = 40 mg SC daily, *BMI* Body Mass Index, *CrCl* Creatinine clearance, *IQR* Interquartile range, *INR* International Normalized Ratio, *Hgb* Hemoglobin, *Padua score* Padua Prediction Score for Risk of VTE

^a^n (%)

^b^Median (interquartile range)

**Table 2:** Concomitant treatments with enoxaparin

Characteristic	Reduced dose (*n* = 124)	Standard dose (*n* = 1,006)	*P*-value
NSAIDs ^a^	21 (16.9)	208 (20.7)	0.41
Antiplatelet ^a^	14 (11.3)	151 (15)	0.34
Hormonal therapy^a^	1 (0.8)	18 (1.8)	0.71
Chemotherapy ^a^	5 (4)	73 (7.3)	0.26
Use of IPC during admission^a^	23 (18.5)	162 (16.1)	0.52

*NSAIDs* Non-steroidal Anti-inflammatory Drugs, *IPC* Intermittent pneumatic compression, *DVT* Deep Vein Thrombosis, *PE* Pulmonary Embolism, *MI* Myocardial Infarction

^a^n(%)

**Fig. 2:**
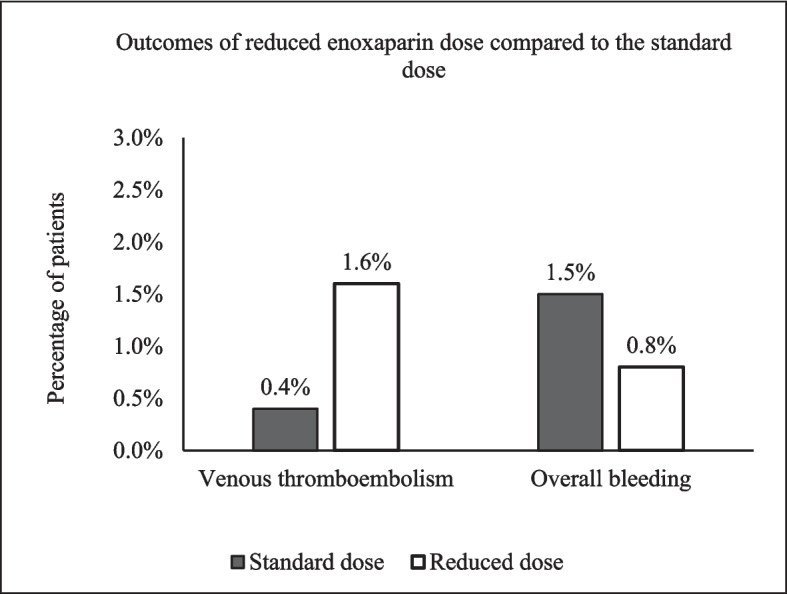
Incidence of overall bleeding and venous thromboembolism by enoxaparin dose

Overall, 16 patients (1.4%) developed bleeding, and six patients (0.53%) developed VTE while on enoxaparin. Bleeding occurred in one patient in the reduced dose group compared to 15 patients in the standard dose group (0.8% vs 1.5%, *p* > 0.99), while two patients in the reduced group had VTE compared to four patients in the standard dose group (1.6% vs 0.4%, *p* = 0.13) (Fig. 2)*.* Of the six VTE events, two were PEs, three were proximal DVTs, and one was distal DVT. In the reduced dose group, the bleeding events were major, while in the standard dose group, five patients had CRNMB, and nine patients had major bleeding. Three patients in the reduced group had the composite unfavourable outcome (bleeding or VTE) compared to 18 patients in the standard dose group (2.4% vs 1.8%, *p* = 0.5).

**Fig. 3:**
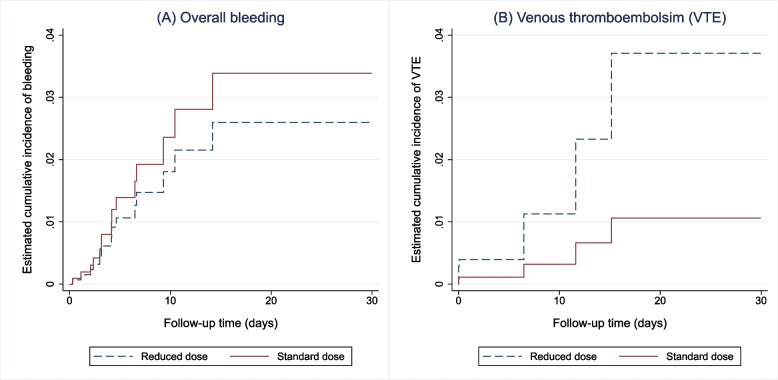
Cumulative incidence function of overall bleeding (**A**) and venous thromboembolism (**B**)

After weighting by the inverse probability of treatment, the absolute standardised differences in Padua risk score, critical illness, and history of bleeding between the two groups were less than 0.1, indicating a balanced distribution of these confounders between the two groups in the weighted population (Supplementary Appendix: Table 2). The IPTW analysis showed that, compared to dose reduction, the standard dose was not significantly different in terms of overall bleeding (OR 1.4, 95% CI 0.18–10.75, *p* = 0.74), VTE (OR 0.3, 95% CI 0.05–1.81, *p* = 0.19), or the composite unfavourable outcome was not different between the standard dosing and the reduced dosing strategies (OR 0.74, 95% CI 0.2–2.75, *p* = 0.66). Similarly, the survival analysis demonstrated that the standard dose was not associated with different risk of overall bleeding (sHR 1.31, 95% CI 0.17–9.92, *p* = 0.79) or VTE (sHR 0.28, 95% CI 0.05–1.71, *p* = 0.17), compared to the reduced dose (Fig. 3).

## Discussion

In this retrospective cohort study, we compared the incidence of bleeding and VTE in underweight patients receiving reduced doses of enoxaparin (< 40 mg daily) versus the standard dose (40 mg daily) for VTE prophylaxis. We did not find a significant difference in the incidence of bleeding and VTE between the two regimens. The lack of significant difference between the two groups is probably due to the small number of events and the small number of patients in the reduced dose group (124 patients).

The incidence of bleeding in underweight patients who received standard-dose enoxaparin in our study was 0.8%, which is lower than the incidence in similar previous studies [14–17]. While Dybdahl et al. have attributed the high bleeding rate to the setting of a level- 1 trauma centre in their study, other studies have also reported higher rates compared to our study [14, 16, 17]. Thus, our study does not exclude a reduction in the incidence of bleeding with dose reduction among patients at higher baseline risk. On the other hand, the overall incidence of VTE in our study (0.53%) was consistent with these studies [14–17]. Given the low baseline rate of VTE among hospitalised patients receiving enoxaparin for prophylaxis in this study and previous studies [14–17], it is unlikely that future studies will find a significantly increased risk of VTE with a dose reduction of enoxaparin.

The standard dose of enoxaparin is associated with higher levels of anti-Xa in underweight patients. A single standard prophylactic dose (40 mg) of enoxaparin in low-weight women (< 45 kg) and low-weight men (< 57 kg) was associated with an increase in anti-factor Xa exposure up to 52% and 27%, respectively, when compared to normal weight control subjects [18]. Rojas et al. also demonstrated an inverse correlation between anti-Xa levels and body weight in patients weighing less than 55 kg receiving enoxaparin 40 mg daily, with the highest anti-Xa levels in the subgroup of patients weighing less than 45 kg [7]. However, as with most surrogate markers, large study populations with high likelihoods of the outcome are needed to demonstrate the reflection of changes in anti-Xa on bleeding and thrombosis.

The evidence for reducing enoxaparin dose in underweight patients comes from several studies. Yam et al. suggested that using a lower dose of enoxaparin for VTE prophylaxis may be sufficient to achieve the goal of peak anti-Xa levels in underweight patients without increased risk of thrombosis [11]. Another cohort study concluded that enoxaparin 30 mg daily is likely to provide adequate thromboprophylaxis in underweight females [12]. However, this evidence is based only on a surrogate marker rather than clinical outcomes, as provided by our study.

Despite the statistical insignificance in our study, patients who received a reduced dose of enoxaparin had a numerically lower incidence of major bleeding. These findings are consistent with three retrospective studies that demonstrated a nonsignificant trend of increased major bleeding in the standard group compared to the reduced dose [14, 17, 19]. The lack of significance in our study may be attributed to the overall low bleeding rates, reflecting a low statistical power to prove a difference. Indeed, a multicentre retrospective study reported that the standard dose had higher odds of causing major bleeding compared to the reduced dose after adjustment for age, gender, and admission haemoglobin (OR 4.73, 95% CI 1.05—21.34) [16]. On the other hand, a retrospective study of patients weighing < 45 kg did not show any trend of reduction in major or CRNM bleeding between 30 mg/day and 40 mg/day of enoxaparin for VTE prophylaxis, which can be attributed to the older age and lower renal function in the reduced dose group with no adjustment for this source of confounding in the analysis [15]. These imbalances may have offset the benefit of dose reduction.

To our knowledge, this is the largest multicentre study to address this important clinical question. However, the study has some limitations. Similar to previous studies, the retrospective design might have introduced bias to the results. Despite using IPTW analysis to adjust for potential confounders, the risk of unobserved confounding still exists. In addition, the study probably did not have enough statistical power due to the low number of outcome events. We did not investigate the impact of specific reduced doses of enoxaparin (e.g., 20 mg and 30 mg). Also, most patients did not have anti-Xa levels since measurement of anti-Xa levels for underweight patients on enoxaparin is not the standard practice in our health system. Thus, we could not investigate the appropriateness of anticoagulation with reduced doses in our cohort. Lastly, we defined underweight as BMI ≤ 18.5 kg/m^2^ or actual body weight ≤ 57 kg; different definitions of underweight may have led to different results.

Based on the observed incidence of bleeding in the two groups in this study, a study of around 7500 patients is required to achieve 80% power at a significance level of 5%, assuming equal study groups. Therefore, future studies that evaluate the reduction in bleeding risk with dose reduction should aim to include patients from national registries or large databases to achieve adequate statistical power. Alternatively, future studies may include patients at higher risk of bleeding to have higher incidence of bleeding and subsequently higher statistical power.

## Conclusions

A variety of VTE prophylaxis regimens are used in underweight hospitalized patients, and pre-emptive dose reduction is uncommon. In this study, there was no significant difference in the incidence of bleeding or VTE between the reduced dose and the standard dose of enoxaparin in this population. The low event rates in this study limit the generalizability of our findings to patients at higher risk of VTE or bleeding until further studies explore the safety and efficacy of dose reduction in high-risk populations.

## Supplementary Information

Supplementary Material 1.

## Data Availability

The datasets used and/or analysed during the current study are available from the corresponding author on reasonable request.

## References

[1] Kahn SR, Lim W, Dunn AS, Cushman M, Dentali F, Akl EA, et al. Prevention of VTE in nonsurgical patients: Antithrombotic Therapy and Prevention of Thrombosis, 9th ed: American College of Chest Physicians Evidence-Based Clinical Practice Guidelines. Chest. 2012;141:e195S-e226S. 22315261 10.1378/chest.11-2296 PMC3278052

[2] Barbar S, Noventa F, Rossetto V, Ferrari A, Brandolin B, Perlati M, et al. A risk assessment model for the identification of hospitalized medical patients at risk for venous thromboembolism: the Padua Prediction Score. J Thromb Haemost. 2010;8:2450–7. 20738765 10.1111/j.1538-7836.2010.04044.x

[3] Wolberg AS, Rosendaal FR, Weitz JI, Jaffer IH, Agnelli G, Baglin T, et al. Venous thrombosis Nat Rev Dis Primer. 2015;1:15006. 10.1038/nrdp.2015.6 27189130

[4] Enoxaparin. Lexi-Drugs. Lexicomp Online. Hudson, Ohio. Wolters Kluwer Clinical Drug Information, Inc.; 2022. Available from: http://online.lexi.com/lco/action/home. Cited 2022 Jul 24.

[5] Levin A, Ben-Artzi M, Beckerman P, Haber G, Varon D, Ben-Yehuda A, et al. Factors Associated with Bleeding in Elderly Hospitalized Patients Treated with Enoxaparin Sodium: A Prospective, Open-Label. Observational Study Drugs Aging. 2009;26:77–85. 19102516 10.2165/0002512-200926010-00006

[6] Abbate R, Gori AM, Farsi A, Attanasio M, Pepe G. Monitoring of low-molecular-weight heparins in cardiovascular disease. Am J Cardiol. 1998;82:33L-36L. 9737479 10.1016/s0002-9149(98)00111-8

[7] Rojas L, Aizman A, Ernst D, Acuña MP, Moya P, Mellado R, et al. Anti-Xa Activity After Enoxaparin Prophylaxis In Hospitalized Patients Weighing Less Than Fifty-Five Kilograms. Thromb Res. 2013;132:761–4. 24521789 10.1016/j.thromres.2013.10.005

[8] Sebaaly J, Covert K. Enoxaparin Dosing at Extremes of Weight: Literature Review and Dosing Recommendations. Ann Pharmacother. 2018;52:898–909. 29592538 10.1177/1060028018768449

[9] Schünemann HJ, Cushman M, Burnett AE, Kahn SR, Beyer-Westendorf J, Spencer FA, et al. American Society of Hematology 2018 guidelines for management of venous thromboembolism: prophylaxis for hospitalized and nonhospitalized medical patients. Blood Adv. 2018;2:3198–225. 30482763 10.1182/bloodadvances.2018022954 PMC6258910

[10] Papazachariou A, Papadakis JA, Malikides V, Nikiforou A, Alexiadou D, Malikides O, et al. Monitoring anti-Xa levels in elderly medical patients undergoing thromboprophylaxis: A prospective cohort study. Geriatr Gerontol Int. 2024;24:587–94. 38705573 10.1111/ggi.14891

[11] Yam L, Bahjri K, Geslani V, Cotton A, Hong L. Enoxaparin Thromboprophylaxis Dosing and Anti-Factor Xa Levels in Low-Weight Patients. Pharmacother J Hum Pharmacol Drug Ther. 2019;39:749–55. 10.1002/phar.2295 31112313

[12] Hakeam HA, Al Duhailib Z, Alsemari M, Alwaibah RM, Al Shannan MF, Shalhoub M. Anti-Factor Xa Levels in Low-weight Surgical Patients Receiving Enoxaparin for Venous Thromboembolism Prophylaxis: A Prospective Cohort Study. Clin Appl Thromb. 2020;26:107602962093119. 10.1177/1076029620931194 PMC7427004 32559127

[13] Schulman S, Kearon C. Definition of major bleeding in clinical investigations of antihemostatic medicinal products in non-surgical patients. J Thromb Haemost. 2005;3:692–4. 15842354 10.1111/j.1538-7836.2005.01204.x

[14] Kaylor DM, Wade RM, Chappell KB, Niemann MH, VanArsdale VM. Venous Thromboembolism Prophylaxis with Enoxaparin Versus Unfractionated Heparin in Patients with Low Body Weight. Plasmatology. 2023;17:26348535231156850.

[15] Dybdahl D, Walliser G, Pershing M, Collins C, Robinson D. Enoxaparin Dosing for Venous Thromboembolism Prophylaxis in Low Body Weight Patients. Clin Med Insights Blood Disord. 2019;12:1179545X1986381. 10.1177/1179545X19863814 PMC6637836 31360075

[16] Buckheit D, Lefemine A, Sobieraj DM, Hobbs L. Venous Thromboembolism Prophylaxis in Underweight Hospitalized Patients. Clin Appl Thromb. 2021;27:107602962110187. 10.1177/10760296211018752 PMC8182211 34080451

[17] Knox H, Edwin SB, Giuliano C, Paxton RA. Venous Thromboembolism Prophylaxis in Low Body Weight Critically Ill Patients. J Intensive Care Med. 2024;39:493–8. 38111295 10.1177/08850666231217693

[18] Lovenox (enoxaparin) [prescribing information]. Bridgewater, NJ: Sanofi-Aventis US LLC. 2021. Available from: https://www.accessdata.fda.gov/drugsatfda_docs/label/2021/020164s129lbl.pdf. Cited 2023 Jul 2.

[19] Nemeth A, Isherwood M. Safety and Effectiveness of Reduced Dose Versus Standard Dose Enoxaparin Venous Thromboembolism Prophylaxis in Underweight Medically Ill Patients. Hosp Pharm. 2023;58:178–82. 36890953 10.1177/00185787221123220 PMC9986578

