# Supplementary material for: Enoxaparin dosing for venous thromboembolism prophylaxis in hospitalized underweight adult patients: a retrospective cohort study
Source: Thromb J. 2025 May 7;23:45. doi: 10.1186/s12959-025-00716-w (PMC12060466; doi:10.1186/s12959-025-00716-w)
Supplement: Supplementary file 1 — Supplementary Material 1. [file 12959_2025_716_MOESM1_ESM.docx]

Table 1: Standardized differences between the two groups **before** inverse probability of treatment weighting (IPTW)

|  | Mean in standard dose | Mean in reduced dose | Standardized difference |
| --- | --- | --- | --- |
| Padua Score | 4.26 | 4.24 | 0.01 |
| Critical illness | 0.07 | 0.16 | -0.293 |
| History of bleeding | 0.03 | 0.03 | -0.038 |

Table 2: Standardized differences between the two groups **after** IPTW

|  | Mean in standard dose | Mean in reduced dose | Standardized difference |
| --- | --- | --- | --- |
| Padua Score | 4.26 | 4.25 | 0.005 |
| Critical illness | 0.08 | 0.08 | -0.001 |
| History of bleeding | 0.03 | 0.03 | -0.019 |
